# Excitation/Inhibition Balance as a Mechanistic Link Between Sleep Disturbance and Autism Spectrum Disorder: A Bidirectional Framework

**DOI:** 10.31083/AP52264

**Published:** 2026-08-13

**Authors:** Yuhan Wu, Di Wu, Wei Zhong, Jun Ma

**Affiliations:** ^1^Department of Developmental and Behavioral Pediatrics, Shanghai Children’s Medical Center, School of Medicine, Shanghai Jiao Tong University, 200127 Shanghai, China

**Keywords:** autism spectrum disorder, sleep, excitation/inhibition balance

## Abstract

Sleep disturbances are highly prevalent in autism spectrum disorder (ASD), yet their significance may extend beyond that of a common co-occurring condition. Growing evidence suggests that excitation/inhibition (E/I)-related network dysregulation may provide a valuable framework for understanding the association between sleep disturbances and variability in core ASD symptom expression. This review synthesizes findings from polysomnography, actigraphy, circadian rhythm research, neuroimaging, electrophysiological studies, and animal models to examine how E/I-related vulnerability in ASD may contribute to impaired sleep initiation and maintenance. It further explores how sleep and circadian disruption may, in turn, influence daytime symptom expression through alterations in synaptic homeostasis and network rebalancing. By proposing a bidirectional framework, this review aims to integrate current evidence on sleep-related symptom variability in ASD and to inform future longitudinal, mechanistic, and biomarker-driven research.

## Main Points

1. Sleep disturbance in autism spectrum disorder (ASD) may represent a clinically and mechanistically relevant dimension, rather than merely a secondary co-occurring condition.

2. Excitation/inhibition (E/I)-related network dysregulation may provide a useful framework for interpreting reciprocal associations between sleep problems and variability in ASD symptom expression.

3. Baseline alterations in inhibitory regulation, network synchrony, and circadian regulation may increase vulnerability to impaired sleep initiation and maintenance in ASD.

4. Poor sleep may be associated with greater sensory dysregulation, repetitive behaviors, and cognitive inflexibility, potentially through altered synaptic homeostasis and impaired overnight network recalibration.

5. Future multimodal longitudinal and interventional studies should integrate objective sleep, circadian, E/I-related, and symptom measures to test this candidate bidirectional framework within the same individuals.

## 1. Introduction

Autism spectrum disorder (ASD) is a heterogeneous neurodevelopmental condition characterized by persistent difficulties in social communication and interaction, together with restricted and repetitive patterns of behavior, interests, or activities [1]. Although prevalence estimates vary across regions and surveillance systems, ASD has become increasingly common worldwide, with reported rates approaching 1.5% in several developed countries and exceeding this level in some settings [2,3].

Sleep disturbance is among the most common co-occurring problems in ASD. Approximately 40%–93% of children with ASD experience sleep-related difficulties, including prolonged sleep latency, frequent nocturnal awakenings, reduced sleep efficiency, and circadian dysregulation, with rates consistently higher than those observed in typically developing children [4,5,6,7,8,9]. These difficulties have traditionally been interpreted as secondary features of ASD or as consequences of associated factors, such as anxiety, gastrointestinal discomfort, sensory over-responsivity, family routines, or the sleep environment [10,11]. This view, however, is increasingly incomplete. Emerging evidence suggests that sleep disturbance is closely linked to the neurobiology and clinical expression of ASD. Sleep abnormalities may reflect underlying neural dysfunction and may also contribute to day-to-day variation in core symptom expression by influencing brain development, synaptic plasticity, and network stability [12,13,14].

One influential framework for understanding the relationship between sleep disturbance and ASD is disruption of excitation/inhibition (E/I) balance. Since Rubenstein and Merzenich [15] first proposed this hypothesis in 2003, it has remained a major model for interpreting the neurobiology of ASD. The original hypothesis suggested that relatively increased excitatory glutamatergic transmission, together with relatively reduced inhibitory GABAergic signaling, may shift circuit dynamics toward excitation, resulting in heightened circuit excitability and a reduced signal-to-noise ratio. These changes may disrupt information processing and network connectivity, thereby contributing to the cognitive and behavioral phenotype of ASD [15]. More recent work, however, suggests that E/I balance should not be conceptualized as a fixed ratio that is uniformly elevated across all brain regions or across all individuals. Rather, it is better understood as a dynamic network property that is state-dependent and circuit-specific [16]. This perspective is particularly relevant to sleep, because the sleep–wake cycle is closely coupled to synaptic plasticity and the homeostatic regulation of neural networks. Wakefulness is generally associated with net synaptic potentiation, whereas sleep is thought to support synaptic reorganization, network recalibration, and homeostatic recovery [17]. Within this framework, sleep disturbance is not merely a parallel feature of ASD, but may be linked with pre-existing vulnerabilities in cortical signal regulation and brain-state transitions.

The relationship between sleep disturbance and ASD symptom expression may therefore be considered within an E/I-related network-state framework. Impaired inhibitory regulation, altered sensory gating, and dysregulated arousal may increase vulnerability to hyperarousal, difficulty initiating sleep, and disrupted sleep architecture [18,19]. Conversely, persistent sleep disruption may be associated with altered synaptic homeostasis, plasticity-related remodeling, and impaired network recalibration, which may in turn contribute to daytime behavioral variability and cognitive difficulties [20]. Although direct mechanistic evidence remains incomplete, findings from clinical observation, neurophysiology, neuroimaging, and genetics collectively support a proposed bidirectional framework. In this framework, E/I-related vulnerability in ASD may contribute to difficulties in sleep initiation and maintenance, whereas sleep disruption may further affect E/I-related network stability and day-to-day symptom variability.

## 2. Sleep Problems in Children With ASD

### 2.1 Subjective Sleep Assessment and Clinical Sleep Phenotypes

Sleep disturbance in children with ASD is not limited to difficulty falling asleep, but involves abnormalities across multiple domains, including sleep duration, sleep continuity, circadian regulation, and sleep microarchitecture. Because many individuals with ASD show sensory over-responsivity, difficulty adapting to unfamiliar environments, and limited tolerance of medical monitoring procedures, earlier research and clinical screening have relied heavily on caregiver reports and questionnaire-based subjective assessments. Among these tools, the Children’s Sleep Habits Questionnaire has been one of the most widely used. Findings from these measures consistently indicate a high prevalence of sleep-onset difficulties, frequent nocturnal awakenings, reduced total sleep time, and parasomnias in children with ASD, often accompanied by bedtime resistance, sleep-related anxiety, and daytime sleepiness or fatigue [6,21,22,23]. However, although subjective scales are valuable for identifying clinically relevant sleep complaints, they have limited ability to characterize sleep architecture and provide little insight into the underlying neurophysiological mechanisms.

### 2.2 Objective Sleep Architecture

With the increasing use of objective methods such as polysomnography, sleep electroencephalography, and actigraphy in ASD research, the structural features of sleep abnormalities have become more clearly characterized. Current evidence suggests that the most commonly reported macrostructural sleep alterations in children with ASD include prolonged sleep latency, reduced sleep efficiency, and increased sleep fragmentation [22,24]. These abnormalities also appear to have clinical relevance, as they have been associated with daytime inattention, repetitive behaviors, and poorer social responsiveness [13,25,26].

Beyond broad alterations in sleep continuity and efficiency, several studies have described changes in sleep microstructure, including a lower proportion of rapid eye movement sleep, prolonged REM latency, reduced slow-wave sleep, and decreased sleep spindle density during non-rapid eye movement sleep [12,27,28,29]. Among these features, sleep spindle abnormalities may be particularly relevant to E/I dysregulation. Sleep spindles are generated within thalamocortical circuits, with rhythmic coordination involving the thalamic reticular nucleus playing a central role, and are closely related to sensory gating, sleep continuity, and memory consolidation [30,31]. Reduced spindle density and shorter spindle duration may therefore reflect impaired inhibitory regulation within thalamocortical networks in children with ASD, potentially contributing to less effective filtering of sensory input. This mechanism may help explain why some individuals with ASD remain unusually vulnerable to weak environmental stimuli during sleep, resulting in frequent microarousals and fragmented sleep. The major sleep abnormalities discussed above and their potential E/I-related interpretations are summarized in Table 1 (Ref. [4,5,6,8,12,14,17,20,22,24,27,28,29,30,31,32,33,34]).

**Table 1.** **Major sleep abnormalities in ASD and their potential E/I-related mechanisms**.

Sleep abnormality	Representative findings in ASD	Potential E/I-related interpretation	Representative evidence
Prolonged sleep latency	Difficulty initiating sleep is frequently reported in children with ASD.	May reflect hyperarousal, weakened inhibitory regulation during sleep onset, and impaired transition from wake-related cortical activity to sleep-related network states.	[4,5,6,22,24]
Sleep fragmentation and reduced sleep efficiency	Frequent nocturnal awakenings, reduced sleep continuity, and lower sleep efficiency are commonly described.	May reflect unstable thalamocortical regulation, reduced sensory filtering during sleep, and impaired maintenance of NREM sleep stability.	[22,24,30,31]
Reduced sleep spindle activity	Lower spindle density or atypical spindle features have been reported in some ASD cohorts.	May reflect altered thalamocortical coordination and impaired inhibitory gating through TRN-related circuits.	[27,30,31]
Altered slow-wave activity and sleep homeostasis	Reduced or atypical slow-wave sleep, altered sleep pressure, or abnormal homeostatic regulation have been described in some studies.	May reflect disrupted synaptic homeostasis, incomplete overnight recalibration, and altered sleep-dependent regulation of network excitability.	[17,20,28,29]
REM sleep abnormalities	Reduced REM proportion or prolonged REM latency has been reported in parts of the ASD sleep literature.	May reflect altered sleep-state organization and broader developmental or neuromodulatory dysregulation, although the relationship with E/I balance is less specific.	[12,28,29]
Circadian dysregulation and irregular sleep–wake rhythm	Delayed sleep timing, eveningness, irregular sleep–activity rhythms, and circadian dysregulation are reported in ASD.	May modify arousal regulation, sleep–wake state transitions, and the timing of sleep-dependent E/I-related network rebalancing.	[8,14,32,33,34]

ASD, autism spectrum disorder; E/I, excitation/inhibition; NREM, non-rapid eye movement; TRN, thalamic reticular nucleus; REM, rapid eye movement.

### 2.3 Longitudinal Associations Between Sleep Disturbance and ASD Symptoms

Some longitudinal studies support an association between early sleep problems and later ASD risk or greater symptom burden. Prospective birth-cohort studies in the general population have shown that more frequent nocturnal awakenings during infancy are associated with higher scores on later ASD screening measures [35]. Longitudinal studies of infants at familial high risk for ASD have similarly reported that poorer nighttime sleep quality or greater difficulty initiating sleep is associated with subsequent ASD diagnosis, greater symptom severity, and atypical neurodevelopmental trajectories [36,37]. However, this relationship is not entirely consistent. Large population-based studies have found that although sleep problems in early childhood are associated with later autistic traits, these associations are substantially attenuated, or may even disappear, after adjustment for baseline autistic traits. In contrast, earlier autistic traits appear to predict later sleep problems more consistently [38,39]. Overall, the current evidence does not support viewing early sleep problems as an independent predictor of later core ASD symptom severity. It does, however, indicate that sleep abnormalities are not merely peripheral features, but are closely and persistently linked to the behavioral phenotype and neurodevelopmental trajectory of ASD. This underscores the need to examine, at a mechanistic level, how sleep disturbance is associated with ASD symptom expression through specific neural circuits and state-regulatory processes. Complementary systematic review evidence also links sleep quality with psychological outcomes in ASD, further supporting the relevance of sleep disturbance to daytime functioning and symptom expression [40].

### 2.4 Sleep Timing and Circadian Context in ASD

Sleep disturbance in ASD includes changes in sleep timing, in addition to alterations in sleep continuity and sleep microstructure. Prolonged sleep latency, irregular sleep schedules, and nocturnal awakenings may, in some individuals, partly reflect circadian misalignment. The sleep–wake cycle is regulated by homeostatic sleep pressure and circadian signals coordinated by the suprachiasmatic nucleus, including daily rhythms in activity, melatonin secretion, and sleep propensity [8,41].

This timing component is relevant to the E/I framework. Sleep-dependent synaptic homeostasis, thalamocortical rhythm generation, and overnight network rebalancing depend not only on sleep quantity and architecture, but also on sleep occurring at an appropriate biological time. When sleep is delayed or fragmented, these restorative processes may be less efficient. Sleep-related changes in cortical excitability, inhibitory regulation, and next-day symptom expression may therefore reflect both sleep architecture and circadian timing. Recent Mendelian randomization evidence has linked circadian rhythm traits and sleep patterns with ASD and ADHD, supporting the relevance of sleep timing to neurodevelopmental phenotypes while leaving the underlying mechanisms to be clarified [32]. Circadian regulation also extends beyond the suprachiasmatic nucleus. Clock-related activity has been described in brain regions involved in cognition, reward, memory, and emotional regulation, including the prefrontal cortex, striatum, hippocampus, and amygdala [42]. This is relevant to ASD because local circadian disruption may influence cognitive and behavioral functions even when gross sleep duration is not the primary abnormality.

Circadian biology may also intersect with ASD-related genetic mechanisms. SH3 and multiple ankyrin repeat domains 3 (*SHANK3*) is particularly relevant because *Shank3* deficiency has been associated with altered sleep regulation and circadian transcriptional changes, and *Shank3*-deficient rats show early-life sleep disruption in an ASD-related preclinical model [43,44]. These findings suggest that some ASD-related sleep phenotypes may involve both synaptic/E/I vulnerability and circadian dysregulation.

Sleep timing and rest–activity rhythms can complement conventional measures of sleep duration and sleep efficiency. Actigraphy can capture habitual sleep timing, rhythm regularity, and day-to-day variability in the home environment. Chronotype may also be important, especially in adolescents and adults, in whom sleep-onset difficulty may reflect eveningness or delayed circadian phase. Recent studies on circadian preference and family circadian synchronization further support the relevance of sleep timing in ASD [33,34]. Together, these findings add a circadian layer to the sleep–E/I–symptom framework. Some ASD-related sleep phenotypes may reflect altered sleep architecture, circadian misalignment, or both. Considering sleep timing alongside E/I-related measures may improve the interpretation of sleep disturbance and daytime variability in ASD symptom expression.

## 3. The Concept of E/I Balance and Its Influence on Brain and Behavior

### 3.1 The Concept of E/I Balance

E/I balance refers to the coordinated relationship between excitatory and inhibitory influences within neural circuits, operating across both magnitude and timing, and enabling neurons to respond effectively to incoming signals while maintaining network stability [45,46]. Previous work has shown that sustained activity in local networks depends on the coordinated recruitment of excitation and inhibition; as excitatory activity increases, inhibitory activity is concurrently engaged to keep overall network activity within a regulated range [47]. E/I balance does not require excitation and inhibition to be strictly equal at every moment. Rather, its defining feature is the preservation of a coordinated relationship between the two within specific circuits and during specific neural events. Accordingly, E/I imbalance may manifest as shifts in the relative proportion, recruitment timing, or spatial extent of excitatory and inhibitory influences, rather than simply as increased excitation or reduced inhibition alone [45,48]. When local circuits fail to recruit sufficient inhibition in parallel with increased excitatory activity, neural networks may become more vulnerable to interference from irrelevant inputs. Conversely, when inhibition is engaged too early, too strongly, or with insufficient flexibility, it may constrain the propagation and updating of information [49,50].

### 3.2 Conceptual Boundaries of E/I Balance and Related Readouts

E/I balance should be distinguished from the indirect measures and functional readouts used to infer E/I-related neural states. In this review, E/I balance refers primarily to the dynamic coordination of excitatory and inhibitory influences within specific neural circuits and behavioral states, rather than to a fixed or globally uniform excitation-to-inhibition ratio [45,46]. Molecular measures such as GABA, glutamate, and glutamine provide information about neurotransmitter systems related to inhibition and excitation, but they do not directly quantify circuit-level E/I balance, particularly when assessed using bulk neurochemical approaches such as magnetic resonance spectroscopy (MRS) [51]. Cellular and circuit-level features, including parvalbumin interneuron function, thalamic reticular nucleus activity, and thalamocortical coordination, are more closely related to mechanisms that regulate E/I balance, although in human ASD studies they are still usually inferred indirectly [31,52,53]. Electrophysiological measures such as gamma oscillations and sleep spindles may reflect network synchrony, inhibitory timing, and sleep-related thalamocortical organization, whereas sensory gating, cognitive flexibility, and symptom variability are better regarded as functional or behavioral manifestations of these neural processes [30,54,55]. Therefore, these measures are discussed here as E/I-related markers or readouts rather than as direct equivalents of E/I balance itself (Table 2, Ref. [13,16,17,18,19,20,26,27,30,31,45,46,48,51,52,53,54,55,56]).

**Table 2.** **Interpreting E/I-related evidence in ASD sleep research**.

Domain	Representative elements	Interpretation within the E/I framework	Key caveat
E/I balance as a framework	Dynamic coordination between excitation and inhibition within specific circuits and brain states [45,46,48]	Provides a conceptual framework for interpreting relationships among sleep disturbance, circuit regulation, and variability in ASD symptom expression.	E/I balance is not a fixed global ratio and cannot be inferred from any single measure.
Neurochemical evidence	GABA, glutamate, and glutamine measured by MRS [51,56]	Suggests alterations in inhibitory and excitatory neurotransmitter systems in at least some individuals with ASD.	Bulk metabolite levels do not directly measure synaptic or circuit-level E/I balance.
Circuit mechanisms	PV interneurons, thalamic reticular nucleus activity, and thalamocortical coordination [18,19,52,53]	Supports a potential role for altered inhibition, sensory filtering, sleep spindle generation, and brain-state regulation.	Most circuit-level mechanisms are inferred indirectly in human ASD sleep studies.
Electrophysiological signatures	Gamma oscillations, sleep spindles, and sleep-related rhythmic activity [27,30,31,55]	May reflect network synchrony, inhibitory timing, thalamocortical organization, and sleep-related recalibration.	These signals are state-, task-, age-, and method-dependent, and are not specific to E/I balance alone.
Sleep-dependent plasticity	Synaptic homeostasis, network recalibration, and restoration of cortical excitability [16,17,20]	Offers a potential mechanism by which disrupted sleep may influence next-day network stability and symptom variability.	Direct evidence integrating sleep, E/I-related measures, and ASD symptom trajectories within the same individuals remains limited.
Behavioral correlates	Sensory gating, sensory burden, repetitive behavioral patterns, and cognitive flexibility [13,26,54]	Represents functional domains through which E/I-related dysregulation may be clinically expressed.	These features are not direct measures of E/I balance and are also shaped by development, co-occurring conditions, medication use, and environmental context.

GABA, gamma-aminobutyric acid; MRS, magnetic resonance spectroscopy; PV, Parvalbumin.

### 3.3 The Influence of E/I Balance on Brain Function

At the level of brain function, E/I balance shapes the stability of cortical activity, the temporal coordination of neural responses, and the rhythmic organization of local networks. Although the most direct evidence has largely come from studies of local circuits in animal models, these findings consistently indicate that the coordinated recruitment of excitation and inhibition is fundamental to information processing and network organization in the brain. In *in vivo* recordings from the rat somatosensory cortex, Okun and Lampl [48] found that excitatory and inhibitory inputs remained closely synchronized during both spontaneous activity and sensory-evoked responses, with correlated changes in amplitude. These findings suggest that cortical responses depend on close coupling between excitation and inhibition as network activity evolves [48]. This coordinated relationship helps constrain the uncontrolled spread of activity and supports the stability and orderly operation of local circuit responses. E/I balance also contributes to the temporal organization of neuronal population activity. Within local cortical networks, fast-spiking interneurons play a major role in regulating gamma-band activity. Using *in vivo* optogenetics, Cardin et al. [53] showed that selective activation of fast-spiking interneurons enhanced gamma-band activity and altered the mode of sensory responses. Sohal et al. [52] further demonstrated that suppression of parvalbumin-positive interneurons weakened gamma oscillations, whereas activation of these cells was sufficient to induce gamma rhythms and improve cortical circuit performance. Overall, these findings indicate that inhibitory interneurons are critical for the synchronization and rhythmic organization of local networks.

### 3.4 The Influence of E/I Balance on Behavior

Because E/I balance is closely linked to information processing and network organization in the brain, disruption of this balance may ultimately be expressed as alterations in cognition and behavior [57]. Available evidence suggests that dysregulation of the relationship between excitation and inhibition within local circuits can alter neuronal population activity and influence higher-order functions, including social behavior, cognitive flexibility, and goal-directed behavior. Yizhar et al. [58] showed that acutely increasing the local E/I ratio in the medial prefrontal cortex of mice impaired cellular information processing and was accompanied by marked alterations in social behavior, suggesting that a local shift in E/I balance can be sufficient to produce behavioral abnormalities in experimental models. This view is further supported by genetic models. In *Cntnap2*-deficient mice, Selimbeyoglu et al. [59] found that reducing the local E/I ratio in the prefrontal cortex could acutely ameliorate social deficits and hyperactivity. E/I regulation is also critically important for executive function. PV interneurons in the prefrontal cortex contribute to the maintenance of working memory and the regulation of cognitive flexibility, and impairment of their function can compromise performance on related tasks [60]. In *Dlx5/6*+/− mice, Cho et al. [55] further demonstrated that abnormalities in prefrontal fast-spiking interneurons were accompanied by reduced task-evoked gamma activity and cognitive rigidity, whereas stimulation at gamma frequency partially restored behavioral flexibility.

**Fig. 1.**
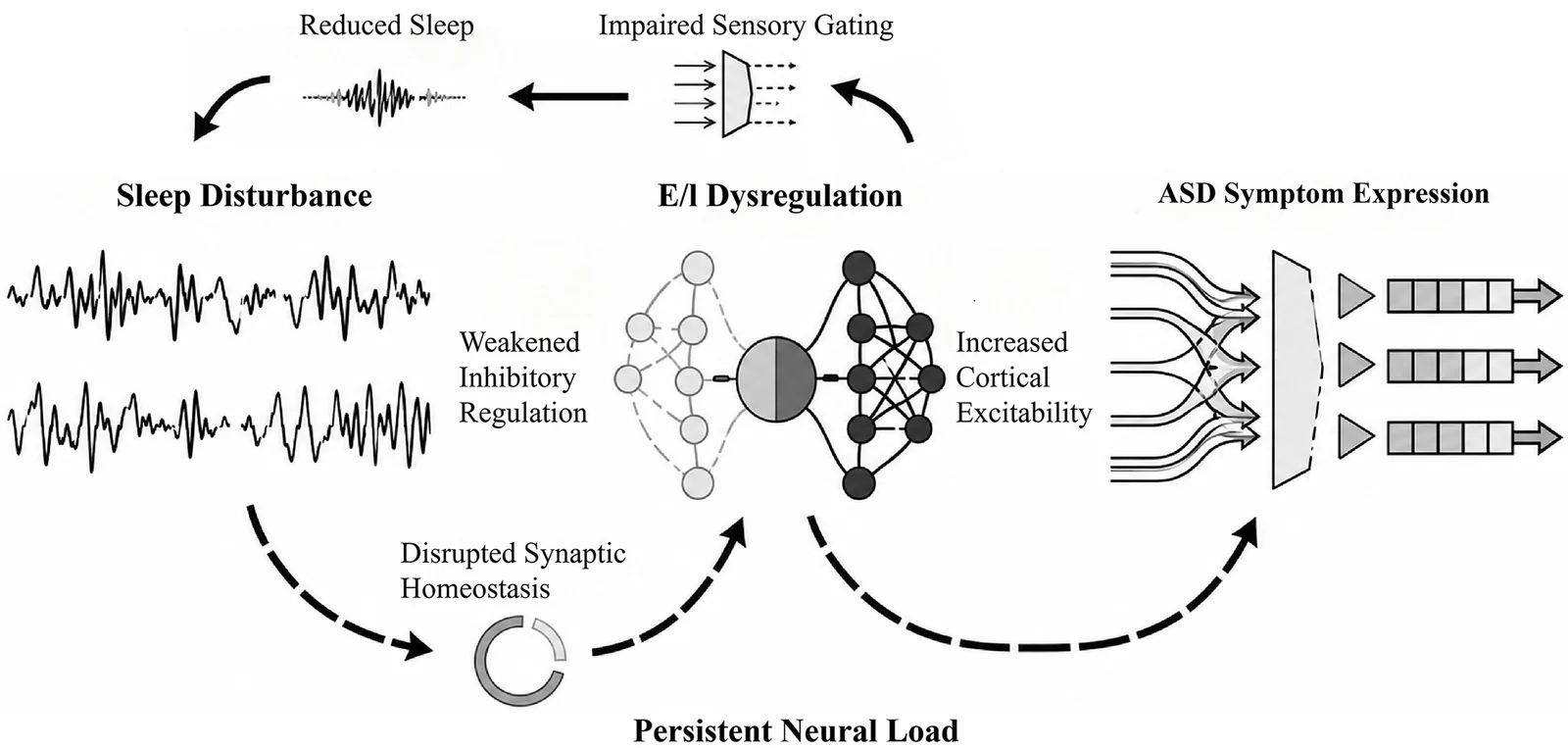
**Proposed bidirectional framework linking E/I balance, sleep disturbance, and variability in ASD symptom expression**. Baseline E/I dysregulation in ASD may weaken inhibitory regulation, increase cortical excitability, impair sensory gating, and destabilize sleep-related rhythms, thereby increasing vulnerability to sleep disturbance. Reduced sleep spindle activity is shown as one representative electrophysiological readout of unstable sleep-related thalamocortical organization. In the reciprocal direction, sleep disturbance may interfere with overnight synaptic homeostasis and contribute to persistent neural load, potentially feeding back into E/I-related dysregulation. This altered network state may be associated with daytime ASD symptom expression across multiple domains, including sensory burden, reduced flexibility, and repetitive or constrained behavioral organization. The figure summarizes a proposed mechanistic framework rather than an established causal pathway.

Taken together, the available evidence supports E/I dysregulation as a plausible mechanistic bridge linking sleep disturbance and symptom expression in ASD. On this basis, we propose the bidirectional framework shown in Fig. 1.

## 4. Sleep Disturbance May Shape Variability in ASD Symptom Expression in Relation to E/I Balance

### 4.1 Sleep-Dependent Rebalancing of E/I-Related Network States

One plausible explanation for the association between sleep disturbance and daytime symptom variability in ASD involves sleep-dependent synaptic homeostasis and the dynamic regulation of E/I-related network states. Studies in wild-type animal models and typically developing populations have shown that cortical excitability and E/I-related network states fluctuate across the sleep–wake cycle [16,61]. During wakefulness, ongoing sensory input and learning-related activity are associated with a progressive increase in cortical excitability as time awake increases. Under conditions of sleep deprivation, related studies have also documented increased cortical excitability, enhanced glutamate-related facilitation, and reduced GABAergic inhibition [61,62,63]. In contrast, sleep, particularly NREM sleep characterized by slow-wave activity (SWA), contributes to the downscaling of synaptic strength and the restoration of network stability, allowing cortical excitability to return to a more physiologically regulated range [64]. At the same time, different classes of inhibitory interneurons show distinct patterns of engagement across NREM and REM sleep, suggesting that sleep-related regulation of E/I balance is a finely tuned process of network rebalancing rather than a simple global increase in inhibition [65].

### 4.2 Disrupted Overnight Rebalancing and Daytime Variability in ASD Symptom Expression

In children with ASD, marked sleep-onset delay or fragmented sleep may interfere with sleep-dependent synaptic homeostasis and network rebalancing [66,67]. Prolonged wakefulness increases sleep pressure and may alter cortical network activity by affecting intracellular chloride homeostasis and the equilibrium potential of GABA_A_ receptor-mediated currents [66]. Persistent difficulty initiating sleep or repeated nocturnal awakenings may therefore be associated with less effective temporal regulation of E/I balance in ASD. Under such conditions, excitatory drive accumulated during wakefulness may not be adequately downscaled during sleep, whereas inhibitory control may be only incompletely restored. As a result, cortical networks may remain under a heightened excitatory load the following day, with relatively insufficient inhibition and reduced flexibility of network regulation [66,68,69]. Moreover, the impact of insufficient sleep may extend beyond transient increases in excitability. Recent work suggests that chronic sleep disruption may also weaken GABAergic inhibitory constraint and alter the plasticity gating of excitatory synapses, rendering some neural circuits more vulnerable to sustained excitatory strengthening [70].

This sleep-related disruption of E/I rebalancing may be clinically expressed, at least in part, as reduced precision of sensory encoding and weakened behavioral regulation [71]. Evidence from studies of sensory cortical circuits and ASD animal models suggests that inhibitory circuit dysfunction, particularly impaired fast inhibition and temporal coordination mediated by PV interneurons, may reduce neuronal tuning precision, decrease the signal-to-noise ratio, weaken sensory gating, and disrupt gamma oscillatory synchrony [54,71]. Accordingly, when insufficient sleep further disrupts the overnight restoration of E/I balance, pre-existing vulnerability in sensory selectivity and background-noise suppression in ASD may become more pronounced, potentially contributing to greater sensory burden, hypersensitivity, and gating difficulties [54]. In addition, insufficient overnight rebalancing may diminish the supportive role of sleep in memory consolidation and circuit updating, leaving the brain more likely to preserve existing patterns of connectivity and response rather than adapt flexibly to changing contextual demands [13,72,73]. This interpretation is consistent with clinical features commonly observed in ASD, including intolerance of change, increased repetitive behaviors, and reduced executive functioning.

### 4.3 Sleep Intervention Evidence and Mechanistic Inference

Intervention studies targeting sleep in children with ASD have shown that behavioral sleep education, telehealth-based parent training, and melatonin, either alone or in combination with behavioral approaches, can improve sleep initiation and sleep continuity. Some studies have also reported improvements in externalizing behaviors or caregiver quality of life [74,75,76]. In addition to behavioral and melatonin-based approaches, neuromodulatory interventions such as repetitive transcranial magnetic stimulation (rTMS) have reported concurrent changes in sleep indices and selected daytime symptoms in children with ASD, although these studies have not yet established whether such changes are mediated by measurable restoration of E/I balance [77,78]. However, improvements in sleep indices are not always accompanied by parallel changes in daytime behavior or family functioning [79]. These mixed findings suggest that sleep improvement may be related to state-dependent changes in daytime functioning, but they do not, by themselves, establish whether such changes are mediated by restoration of E/I balance. Interventional studies that integrate objective sleep measures, E/I-related biomarkers, and repeated symptom assessments are needed to test this mechanism more directly.

## 5. Baseline E/I Dysregulation and Vulnerability to Sleep Problems in ASD

### 5.1 Baseline E/I Dysregulation in ASD

GABA and glutamate, the principal inhibitory and excitatory neurotransmitter systems in the central nervous system, provide key molecular substrates for E/I-related neural regulation. Current MRS studies overall suggest the presence of neurotransmitter alterations related to E/I balance in individuals with ASD. Meta-analytic evidence indicates that GABA levels are reduced overall in ASD, particularly in the frontal lobe and when assessed using the GABA/creatine ratio, whereas glutamate levels in the prefrontal cortex tend to be elevated. These findings are consistent with E/I-related dysregulation in at least a subset of individuals with ASD [51,80]. However, Fung and colleagues reported significantly higher GABA concentrations in the left dorsolateral prefrontal cortex of adults with high-functioning ASD than in controls [81]. This finding suggests that GABA alterations in ASD are not uniform in direction, but may instead be shaped by developmental stage, region-specific effects, and the inherent limitations of MRS as a measure of bulk metabolite levels. In addition, 7 T MRS studies have shown increased glutamine levels in the thalamus and right temporoparietal junction in adults with ASD, suggesting that abnormalities in the glutamate–glutamine cycle may also contribute to intrinsic E/I-related dysregulation [56]. Beyond metabolite-based evidence, electrophysiological studies provide functional readouts related to altered E/I regulation in ASD. The generation of gamma oscillations depends on fast feedback inhibition between excitatory pyramidal neurons and inhibitory interneurons. Current evidence suggests that individuals with ASD often show atypical resting-state gamma activity, whereas during visual or auditory tasks, particularly in the 40-Hz auditory steady-state response paradigm, they tend to exhibit reduced gamma power and impaired phase synchrony [82,83,84]. This dissociation, characterized by atypical resting-state activity alongside insufficient task-evoked synchrony, suggests that ASD may involve impaired fast inhibitory feedback and altered local network synchrony, resulting in dysregulated dynamic control of E/I-related network states.

### 5.2 Baseline E/I Imbalance and Sleep Regulation

Baseline E/I-related dysregulation may be relevant to sleep problems because children with ASD may have greater difficulty filtering external stimuli during sleep onset and establishing stable NREM sleep. Under typical conditions, sleep onset is accompanied by a gradual reduction in the brain’s responsiveness to external sensory input and by a transition of thalamocortical activity from wake-related patterns to sleep-related rhythms [85]. When individuals with ASD already show altered inhibitory neurotransmission, heightened sensory responsivity, and impaired local network synchrony, external auditory, visual, or tactile stimuli may be less effectively suppressed during the transition to sleep, and the establishment of thalamocortical sleep rhythms may be more easily destabilized. Clinically, this may manifest as prolonged sleep latency, difficulty initiating sleep, and more frequent nocturnal awakenings [86,87,88]. In this process, thalamocortical sensory-gating circuits, particularly the thalamic reticular nucleus (TRN), may serve as a critical link. As the principal GABAergic inhibitory structure within the thalamus, the TRN plays a central role in sleep spindle generation, sensory filtering, and the coordination of thalamocortical rhythms [31,89]. Animal studies have shown that TRN dysfunction can lead to reduced sleep spindle activity and increased sleep fragmentation [90]. Human studies have likewise reported lower sleep spindle density and atypical spindle characteristics in children with ASD, suggesting disruption of TRN-related thalamocortical sleep rhythms [27,91]. Overall, baseline E/I dysregulation in ASD may promote both sleep-onset and sleep-maintenance difficulties by weakening sensory gating during sleep initiation and destabilizing the processes required for stable NREM sleep.

This mechanism is also indirectly supported by ASD-related genetic models. Animal studies have shown that several high-confidence ASD risk genes are involved not only in synapse formation and the maintenance of E/I balance, but also in sleep regulation. First, *SHANK3*, which encodes a major scaffolding protein of the excitatory postsynaptic density, is essential for synaptic organization and function. *Shank3* deficiency not only produces autism-related abnormalities in social and repetitive behaviors, but is also associated with insufficient inhibitory circuit engagement and E/I dysregulation [92]. *Shank3* knockout models exhibit reduced total sleep time, decreased NREM sleep, and altered responses to sleep deprivation [44,93]. These findings support a link among synaptic scaffolding, E/I regulation, and sleep homeostasis in *Shank3*-related models. Second, fragile X messenger ribonucleoprotein 1 (*FMR1*), the causative gene for fragile X syndrome, is one of the most common genes implicated in monogenic/syndromic forms of ASD. Loss of fragile X messenger ribonucleoprotein (FMRP) leads to abnormal synaptic regulation involving metabotropic glutamate receptor 5, and in both *Drosophila* and mouse models, this is accompanied by impaired sleep homeostasis, abnormal rebound after sleep deprivation, and altered sleep architecture [94,95]. Third, synaptic adhesion molecules such as neurexin 1 (*NRXN1*) and neuroligin 4 (*NLGN4*) are strongly associated with ASD and are involved in synapse formation, maturation, and the coordination of excitatory and inhibitory transmission. In *Drosophila* models, loss of *dnrx-1* leads to fragmented nighttime sleep and circadian rhythm abnormalities, whereas loss of *dNlg4* causes marked sleep deficits through impaired GABAergic transmission [96,97]. These findings collectively suggest that baseline synaptic abnormalities and E/I dysregulation associated with ASD-related genes may themselves impair sleep homeostasis and sleep continuity.

## 6. ASD Heterogeneity and the Sleep–E/I–Symptom Association

ASD encompasses substantial developmental, cognitive, genetic, and clinical heterogeneity, which is highly relevant when E/I balance is used to interpret the association between sleep disturbance and ASD symptom expression. Sleep phenotypes, E/I-related neural measures, and daytime symptom expression may vary according to developmental stage, cognitive and language ability, genetic background, co-occurring conditions, and treatment exposure. Accordingly, the associations among sleep disturbance, E/I dysregulation, and variability in ASD symptom expression may be expressed differently across developmental and clinical subgroups.

Sleep architecture and E/I-related regulation are developmentally dynamic rather than static across age. The developmental transition of GABAergic signaling, maturation of slow-wave activity, and age- and region-specific organization of sleep spindles all vary across developmental periods [27,31,98]. Thus, early thalamocortical maturational delay should not be conflated with chronic network-regulatory abnormalities observed during adolescence or adulthood. Baseline cognitive and language abilities may also affect the validity of sleep assessment and the interpretation of underlying neural phenotypes. Individuals with limited functional language or intellectual disability may have difficulty communicating interoceptive discomfort, pain, or bedtime-related anxiety, increasing reliance on caregiver reports and the risk that sleep disturbance may be misclassified as bedtime resistance, irritability, or nonspecific behavioral dysregulation [10,99]. Atypical spindle profiles have been reported in young children with ASD, and sleep quality has also been linked to language abilities, suggesting that sleep microstructure and sleep phenotypes should be interpreted alongside cognitive and language profiles [91,100]. Genetic background represents another important source of heterogeneity. *SHANK3 *disruption may involve postsynaptic scaffolding defects, altered inhibitory circuit engagement, and sleep–wake instability [44,92]. *FMR1*-related models suggest abnormalities in sleep need, sleep homeostatic responses, and circadian behavioral rhythms [94,101]. *NRXN1* and *NLGN4* variants may affect synaptic adhesion, sleep regulation, and GABAergic transmission, providing additional routes through which synaptic dysregulation may be linked to sleep–wake instability [96,97].

Common psychiatric and somatic co-occurring conditions may impose additional pressure on neural excitability and sleep regulation. Epilepsy and subclinical epileptiform activity are relatively common in ASD and may disrupt sleep continuity and sleep microstructure, particularly thalamocortical rhythms that support sleep spindles and slow-wave activity [102,103]. ADHD and anxiety may contribute to heightened arousal and delayed circadian timing, thereby complicating the interpretation of sleep-onset difficulties in ASD [104,105]. Gastrointestinal symptoms may increase nocturnal discomfort and sleep disruption through pain, autonomic activation, and brain–gut–immune pathways, whereas sensory over-responsivity may make the sleep-onset period especially vulnerable to low-intensity environmental stimuli [105,106]. Medication exposure represents another major source of heterogeneity in clinical and translational ASD sleep research. Melatonin, antipsychotics, stimulants, and antiseizure medications may influence sleep timing, sleep architecture, circadian regulation, and neural excitability [74,107]. Without careful control for medication status, such effects may confound attempts to estimate intrinsic E/I-related neural profiles in ASD.

## 7. Future Directions

The proposed bidirectional framework should be tested in studies that examine sleep, E/I-related measures, and symptom trajectories within the same individuals. At present, much of the evidence comes from partially separate literatures: clinical sleep studies characterize sleep continuity and sleep architecture, neurochemical and electrophysiological studies index E/I-related neural states, and longitudinal studies track developmental or behavioral outcomes. Although these findings provide convergent support for the framework, they do not yet establish the temporal or causal ordering among sleep disturbance, E/I dysregulation, and variability in ASD symptom expression.

Future longitudinal and interventional studies should therefore combine objective sleep assessments, including polysomnography, high-density electroencephalography (EEG), and actigraphy-based rest–activity rhythm monitoring, with E/I-related biomarkers, circadian measures, and repeated assessments of ASD symptoms. Such designs could help determine whether changes in sleep continuity, sleep spindles, slow-wave activity, sleep timing, or rest–activity rhythm regularity precede, accompany, or follow changes in E/I-related neural states and symptom expression. Chronotype, melatonin rhythm, actigraphy-derived rhythm regularity, and clock gene-related markers may further help distinguish sleep-architecture abnormalities from sleep-timing abnormalities.

## 8. Conclusions

E/I balance offers a coherent mechanistic framework for interpreting the association between sleep disturbance and ASD symptom expression. Within this proposed bidirectional framework, baseline E/I dysregulation in ASD, reflected by altered inhibitory regulation, network synchrony, and sensory gating, may increase vulnerability to difficulties in sleep initiation, sleep maintenance, and NREM sleep stability. Conversely, sleep disturbance may interfere with overnight synaptic homeostasis and network rebalancing, potentially feeding back into E/I-related dysregulation and contributing to variability in sensory processing, repetitive behavioral patterns, and cognitive flexibility. Sleep problems in ASD should therefore not be viewed solely as accompanying symptoms, but as a clinically relevant dimension closely associated with neural circuit regulation and behavioral state. By organizing evidence across sleep physiology, neurophysiology, neuroimaging, and ASD-related genetic models, this framework provides a neurobiologically grounded account of the reciprocal association between sleep disturbance and ASD symptom expression. Future longitudinal and interventional studies integrating objective sleep measures, E/I-related biomarkers, and symptom trajectories within the same individuals are needed to clarify the temporal, causal, and state-dependent components of this relationship.

## Abbreviations

ASD, autism spectrum disorder; E/I, excitation/inhibition; PV, parvalbumin; REM, rapid eye movement; NREM, non-rapid eye movement; SWA, slow-wave activity; GABA, gamma-aminobutyric acid; MRS, magnetic resonance spectroscopy; EEG, electroencephalography; TRN, thalamic reticular nucleus; rTMS, repetitive transcranial magnetic stimulation; *SHANK3*, SH3 and multiple ankyrin repeat domains 3; *FMR1*, fragile X messenger ribonucleoprotein 1; *NRXN1*, neurexin 1; *NLGN4*, neuroligin 4.
